# Canine Histiocytic Malignancies—Challenges and Opportunities

**DOI:** 10.3390/vetsci3010002

**Published:** 2016-01-04

**Authors:** Katherine Kennedy, Rachael Thomas, Matthew Breen

**Affiliations:** 1 Department of Molecular Biomedical Sciences, College of Veterinary Medicine, North Carolina State University, Raleigh, NC 27607, USA; kakenne4@ncsu.edu (K.K.); rachael_thomas@ncsu.edu (R.T.); 2 Comparative Medicine Institute, North Carolina State University, Raleigh, NC 27607, USA; 3 Center for Human Health and the Environment, North Carolina State University, Raleigh, NC 27607, USA; 4 Cancer Genetics Program, University of North Carolina Lineberger Comprehensive Cancer Center, Chapel Hill, NC 27599, USA

**Keywords:** cancer, sarcoma, macrophage, dendritic, disseminated

## Abstract

Canine histiocytic malignancies (HM) are aggressive tumors that occur with particularly high frequency in certain breeds including Bernese mountain dogs and flat-coated retrievers. Robust diagnosis of HM commonly utilizes immunohistochemical stains that are broadly ineffective on formalin-fixed tissues; thus the diagnosis is often one of exclusion. Clinical outcomes are generally poor, with frequent metastasis and therapeutic failure lowering overall survival at time of diagnosis to an average of less than two months in the majority of published work. The limited understanding of the molecular mechanisms underlying HM has hindered the development of more effective diagnostic modalities and the identification of therapeutic targets. A potential avenue exists for advancing clinical management of canine cancers through extrapolation from a close counterpart in human medicine. Historically, HM have been compared to the rare and understudied subset of human cancers involving the dendritic lineage, such as dendritic cell sarcoma or Langerhans cell sarcoma. Recent data have now thrown into question the cellular origin of HM, suggesting that the disease may originate from the macrophage lineage. This review summarizes existing knowledge of HM from the clinical, histologic and molecular perspectives, and highlights avenues for future research that may aid the development of novel diagnostic and therapeutic approaches. In turn, a more advanced appreciation of the mechanisms underlying HM should clarify their cellular origin and identify appropriate opportunities for synergistic extrapolation between related canine and human cancers.

## 1. Introduction 

Canine histiocytic malignancies (HM) represent a spectrum of aggressive neoplasms that while relatively infrequent within the global domestic dog population, are associated with a remarkably high rate of mortality in a subset of breeds [1,2]. Clinical signs typically are non-specific and include fever, weight loss, lethargy, inappetence and, if the tumor presents on a limb, the presence of a noticeably enlarging mass [3]. Since the late 1970s HM have been described in the literature using a variety of terms, including malignant fibrous histiocytoma (MFH) and histiocytic sarcoma complex [4,5]. The benign histiocytomas will not be considered as a part of this review, as they are typically self-resolving [6]. More recently, HM have been sub-classified into two primary subtypes, localized and disseminated histiocytic sarcoma. In this scheme, localized histiocytic sarcoma is considered to originate on a limb, or within a single internal organ, commonly the spleen [7]. A more recently recognized hemophagocytic variant arises only in spleen or bone marrow, sharing similar clinical features with localized HM of the spleen [8], and will be considered as a form of localized HM for the purposes of this review. The disseminated form of histiocytic sarcoma is described as a multifocal disease with masses occurring simultaneously at multiple sites (most commonly spleen, lung, liver, and abdominal lymph nodes), and carries a less favorable prognosis [4,9]. Unfortunately, some studies have utilized the words visceral and disseminated interchangeably. For the purposes of this review, disseminated will be utilized to represent the multi-focal disease at time or presentation. The current belief is that these variants represent the early and late stages of a single disease rather than two unique pathological processes [10,11]. 

While infrequent in the general dog population there is a particularly strong breed predisposition to HM in Bernese mountain dogs (BMD) and flat-coated retrievers (FCR), with odds ratios of 45.0 and 62.0 respectively [3]. Other breeds including Rottweilers [12], golden retrievers and Labrador retrievers show slightly elevated risk (odds ratios of 5.0 (golden retrievers and 3.0 Labrador retrievers) [3]. A recent study also showed elevated risk in the Japanese population of Pembroke Welsh corgis (odds ratio of 9.7), although this was not evident in this breed within other geographical regions [3]. Interestingly, the two most highly predisposed breeds exhibit distinct patterns of disease presentation [9]. The FCR is most commonly affected with the localized variant of HM, accounting for up to 36% of all malignant tumors seen in the breed [5]. As many as 60% of the tumors affecting the spleen represent the highly aggressive hemophagocytic phenotype [13]. Overall, these tumors are responsible for up to 46% of all deaths in the FCR population, and contribute strongly to the nearly two-year reduction in lifespan of the FCR compared to other breeds [1]. In contrast, the disseminated form is more prevalent in the BMD, accounting for up to 64% of all malignant tumors in the breed [1]. These tumors contribute to as many as 50% of deaths in the BMD, and a ~three-year reduction in lifespan [1]. HM therefore have a tremendous impact on the welfare and longevity of these two breeds, and there is a clear need to improve options for clinical management of the disease.

## 2. Clinical Outcomes of Canine HM

Clinical outcomes for HM are extremely poor [1,7], with an overall mean survival after diagnosis of only 49 days (median survival 30 days) [14]. Unfortunately, due to the nature of the clinical signs, it is currently not possible to determine how long these patients have actually had the disease. Treatment typically comprises chemotherapy, commonly utilizing lomustine (CCNU), with or without tumor resection and adjuvant radiotherapy [2,13]. This strategy is rarely successful, since HM rapidly acquire drug resistance resulting in a short-lived response in both single agent and multi-modal treatments [15,16,17]. Using single agent CCNU, only 46% of cases will demonstrate at least a partial response defined as a reduction of gross tumor size by greater than 50% and less than 100%, with a median duration of only 85 days [17]. More recently, one study of sixteen dogs with localized HM surgically treated with curative intent and adjuvant CCNU demonstrated a median disease free interval of 243 days [18]. However, as a part of inclusion in this study, no dogs presenting with local or distant metastatic lesions were included [18]. Due to the generally dismal response rates, and frequent metastasis at diagnosis most dogs presenting with HM are euthanized within days of the initial diagnosis, emphasizing the need for both an accurate and rapid diagnosis [7]. 

The past three years have seen marked progress in assessing alternative therapeutic options for canine HM, however these have either been performed *in vitro* or as retrospective clinical trials [15,19,20,21]. Initial studies focused on the addition of bisphosphonates to vincristine and doxorubicin-based chemotherapy, finding that the macrophage-depleting adjuvant increased both uptake and cell-arrest *in vitro* [15]. Subsequently, the tyrosine kinase inhibitor dasatinib, which targets the SRC proto-oncogene, non-receptor tyrosine kinase (*SRC*), demonstrated a modest ability to inhibit growth of canine HM cell lines [21]. Most recently two studies have used retrospective data to assess the clinical utility of two additional chemotherapeutic regimens [19,20]. Vinorelbine was found to have a complete response rate, defined as the absence of radiographic evidence of tumor, of 22% (2/9 cases, durations of 771 and 162 days), with an additional 4 cases maintaining stable disease (median time to progression of 61 days), and 3 cases in which only progressive disease was seen [19]. Unfortunately the value of this retrospective study is severely limited by both the number of dogs enrolled, and the fact that these represent a range of breeds, differing anatomical locations at time of presentation, and a wide age range (5.7–13.1 years) [19]. The combination of CCNU and doxorubicin demonstrated at least a partial response in 58% of cases (minimum 30% decrease in tumor size in 5/12 and complete response (no evidence of disease) in 2/12), with an overall median survival time of 185 days [20]. Again, while promising, these studies had limited sample size, wide distribution of dog breeds, and initial presentations affect the significance of these studies. In the future, researchers should move towards prospective clinical trials with a focus on highly affected breeds such as the Bernese mountain dog and flat-coated retriever, so that the resulting data will be able to provide a method for robust comparisons between treatment methodologies. 

## 3. Diagnostic Challenges in Canine HM

### 3.1 Historical Diagnostic Scheme

One of the greatest challenges to the development of more efficacious therapies for HM is the lack of certainty regarding the biological origin of the disease. Morphologically, tumors can appear as sheets of large pleomorphic cells, whorls of spindle cells, or a mixture of the two [4]. Cellular atypia is frequent, and bizarre mitotic figures may be evident [4]. Since these features are common to a number of malignant processes, initial differential diagnoses include synovial cell sarcoma [7], lymphoma and plasmacytoma [22], mast cell tumor [23], and amelanotic melanoma [24]. Due to these similarities, immunohistochemistry (IHC) has been considered a necessary part of the diagnostic scheme in canine HM [12].

The most comprehensive diagnostic scheme for HM has been based on IHC staining with CD1 and CD11c (indicative of cells derived from the dendritic lineage), CD18 and CD45 (pan-leukocyte markers) and MHC II (a marker of antigen-presenting cells, Table 1) [4]. Among these only CD1 and CD11c are specific to HM, while positive staining with each of these five markers is considered diagnostic of HM [7,11]. Aside from the inherent cost incurred in diagnosis, application of this approach is confounded by the fact that CD1 has variable efficacy and CD11c is ineffective on formalin-fixed tissue, which therefore limits its utility outside routine diagnostic service laboratories [11]. Consequently HM commonly represents a diagnosis of exclusion, and many cases are defined simply as consistent with a poorly differentiated round cell neoplasm [11]. In the absence of comprehensive IHC profiling it has been estimated that up to 70% of cases may be classified incorrectly as HM, of which approximately 14% represent disease that generally are more suited to therapy and thus potentially have a more favorable outcome (e.g., lymphoma and plasmacytoma) [22]. The cost and sensitivity/specificity limitations of profiling by IHC have encouraged assessment of alternative strategies. 

**Table 1:** Expression patterns of immunohistochemistry (IHC) markers in canine histiocytic malignancies (HM) and other round cell tumors.

Disease	CD79a [4]	CD3 [4]	MUM1 [23]	Melan-A [24]	c-Kit [23]	CD18 [4]	CD11c * [4]	**CD45** [4]	**MHC II** [22]
Histiocytic malignancy	(–)	(–)	(–)	(–)	(–)	(+)	(+)	(+)	(+)
Lymphoma	(+) B cell	(+) T cell	(+/–)	(–)	(–)	(+)	(–)	(–)	(+) B cell
Amelanotic melanoma	(–)	(–)	(–)	(+)	(–)	(–)	(–)	(–)	(–)
Mast cell tumor	(–)	(–)	(–)	(–)	(+)	(–)	(–)	(–)	(–)
Plasma cell tumor	(+)	(–)	(+)	(–)	(–)	(+)	(–)	(–)	(+/–)

Key: (–) negative (no staining present); (+) positive staining; (+/–) variable staining; * not appropriate for use with formalin-fixed tissue.

### 3.2 Advances in Diagnostic Strategies for Canine HM

Recently the macrophage scavenger receptor CD204 has been shown to be effective for IHC evaluation both of formalin-fixed tissue and cytological smears. Positive staining is highly specific for HM, providing a powerful diagnostic indicator for distinction from other round cell neoplasms such as lymphoma and plasmacytoma [11,25]. This raises questions over the historical opinion that the majority of HM arise from the dendritic lineage, since CD204 is considered to be macrophage-specific [11,25]. Shortly after these findings were published, a second canine HM-specific IHC stain, termed Iba1 (ionized calcium binding adapter molecule 1) was reported to demonstrate robust performance in formalin-fixed tissues, offering another promising diagnostic indicator [24]. While Iba1 does not distinguish between dendritic and macrophage cells, data arising from the use of CD204 present an interesting challenge to the current understanding of the cellular origins of HM [25]. At present there is an insufficient volume of robust data available from which to determine unequivocally whether the true cellular origin of HM is that of the classical dendritic cells as described by Moore, or the more macrophage-like cells described by Kato [3,4]. 

## 4. Current Understanding of the Genomics and Underlying Disease Mechanisms of Canine HM

Molecular characterization of canine HM provides a complementary avenue for advancing diagnostic and therapeutic modalities and for clarifying the biological origin of the disease. To date there has been one study describing genome-wide somatic DNA copy number aberrations in HM, and three focusing on tumor-associated gene expression signatures [5,9,10,26]. Microarray-based DNA copy number profiling identified a high incidence of genomic imbalance among 104 HM cases, which was distributed widely across all dog chromosomes [9]. Among the most recurrent aberrations were deletions of the *CDKN2A/B* and *MTAP* loci on dog chromosome 11q16 (CFA11q16), and the *RB1* tumor suppressor gene on CFA22q11. Interestingly the *MTAP* locus has since been identified as a germline susceptibility locus for HM within the BMD breed using genome-wide single nucleotide polymorphism analysis [27]. Subsequent chromosomal analysis of HM cases revealed extensive structural abnormalities, indicative of substantial genomic reorganization that may reflect the highly aggressive nature of the disease [9]. While providing valuable insight into genomic mechanisms that may contribute to HM pathogenesis, the conclusions of the study were limited by the relatively low-resolution genomic microarray platform available at the time, coupled with the challenges associated with characterization of newly-identified structural chromosome rearrangements. With the advent of higher resolution tools for genomic profiling analyses [28,29,30,31] and increasing access to DNA sequence-based methods for structural analysis of cancer genomes [32], a re-visitation of this approach will likely prove fruitful.

Two of the three gene expression studies of HM were restricted to the FCR breed [5,10]. The first utilized microarray-based techniques to compare transcriptional profiles of localized HM and non-neoplastic splenic control tissue, finding significant tumor-associated deregulation of canonical pathways including cell cycle regulation, DNA replication, and DNA mismatch repair [5]. Additionally, genes associated with tumor motility such as *LUM* and *Col3aI* were found to be significantly up-regulated in visceral and external tumors [5]. The interpretation of this study was confounded by the use of splenic tissue as a control to represent an enriched source of antigen-presenting cells, which interfered with data interpretation for two candidate genes, *SPIc* and *VCAM-1* due to their natural involvement in angiogenesis [5]. 

The second study expanded on findings from the first, performing transcriptional profiling of localized HM cases to determine whether significant differences exist between visceral and limb forms of the disease in the FCR [10]. Key findings included down-regulation of *CCL5* and *CLEC12A* and up-regulation of *C6* in visceral tumors [10]. Given the elevated propensity for metastasis in visceral tumors, the down-regulation of *CCL5* was considered unexpected due to the association of this gene with rapid tumor metastasis and cellular motility [10]. *CLEC12A* down-regulation in visceral tumors was considered more consistent with expected findings, as the gene functions as a negative regulator of granulocyte/monocyte function. It was suggested that that these findings may better account for the increased cellular motility in visceral tumors while the up-regulation of *C6* (complement component 6) may represent the interactions of the innate immune response to the tumor cells [10]. Both these studies, however, were confounded by the limited annotation of genes on the expression microarray platform, which hindered comprehensive interpretation of transcriptional variation between tumor subtypes and control tissues. Moreover, the selection of appropriate control tissues was challenging due to the variety of anatomical sites involved in HM, and there was no additional assessment of dysregulated genes using complementary approaches such as western blotting, or IHC.

The final study took a more mechanistic approach, focusing on two genes, *HMGB1* and *RAGE*, which are required for the development of dendritic cells [26]. In this study, both genes were significantly down-regulated in lung masses compared to non-neoplastic tissue controls in 13 cases of disseminated HM in BMDs, indicative of advanced tumor stage and increased tumor aggression [26]. Unfortunately, the significance of the down-regulation of *HMGB1* in disseminated HM specimens from other anatomical sites could not be shown definitively, due to conflicting results from the use of two independent housekeeping genes [26]. IHC analysis showed that the down-regulation of *HMGB1* and *RAGE* mRNA was reflected in a corresponding decrease in protein levels in tumor specimens when compared to control tissues, in combination with a lack of nuclear localization, demonstrating the potential for cytokine production and release in neoplastic cells. The impact of these findings was hindered by a small sample size, and the investigation of only disseminated HM in the BMD population [26]. 

While these studies provide a useful foundation for the definition of molecular mechanisms underlying the pathogenesis of canine HM, their inherent limitations reinforce the need for alternative strategies. 

## 5. The Comparative Value of Canine HM to Human Medicine

Numerous recent studies have supported the concept of advancing progress in clinical management of cancers through extrapolation of data between counterparts of the same disease in different species. HM has most commonly been compared to human sarcomas of the histiocytic and dendritic lineages, which together represent less than 1% of all hematopoietic and lymphoid neoplasms in people [4,33,34]. Tumors arising from these lineages include dendritic cell sarcomas (DS), as well as classical histiocytic sarcoma and Langerhans cell sarcoma (tumors of a more macrophage-like origin) [34,35]. Given the rarity of histiocytic sarcoma and Langerhans cell sarcoma in people and their shared clinical features, they will be considered together in this review as histiocytic neoplasia (HS) [35]. Treatment for DS and HS are broadly similar, with the majority of patients undergoing surgical resection followed by adjuvant chemotherapy [33,35,36], despite which up to 30% of DS patients will demonstrate metastasis [33] and the majority of patients with HS will succumb to the disease within two years [34,37]. There is a clear need for more targeted therapeutics and the establishment of standardized treatment protocols in HS/DS; however the rarity of these tumors is reflected in the small volume of literature describing their biological and clinical characteristics [36,37]. With the limited number of clinical specimens available for analysis, there are very restricted opportunities to contribute to comparative studies. While it is therefore not currently feasible to use in-depth studies of the human disease to significantly inform the veterinary field, a comprehensive evaluation of canine HM may significantly expand our knowledge of both canine and human forms of the disease, resulting in mutual benefit. 

As with canine HM, robust diagnosis of tumors of the HS/DS lineage in human patients remains challenging. Tumors are diagnosed primarily on the basis of their cellular morphology, ranging from spindle to ovoid shaped cells arranged into whorls or sheets [33,34]. While IHC is also used to assist with diagnosis of HS/DS, the markers used most frequently, such as CD68 and vimentin, are non-specific, leading to the potential for misclassification [34,35,36]. Unfortunately, due to the rarity of these tumors, there have been no concerted efforts to identify more informative IHC markers; thus the newer reagents identified for canine HM, particularly the CD204 and Iba1 markers, may represent a useful asset for the human field. 

Molecular characterization of HS/DS is a key requirement for recognizing underlying disease mechanisms and for improving outcome through targeted clinical management. A degree of conflict exists among the small number of reports describing the genome-wide profiles of HS/DS, with the majority of studies indicating relatively stable profiles in both diseases [37,38], while others show extensive disruption both of DNA copy number and chromosome structure, particularly in DS [39,40]. Unfortunately, the majority of these reports comprise either single case studies [40] or small comparisons of up to five samples [37,39], limiting the ability to extract meaningful conclusions.

Assessment of immunoglobulin heavy chain (IGH) receptor clonality in HS/DS has shown the possibility that some cases of HS/DS may have arisen from a previously existing lymphoid neoplasm, based on IGH clonality in both the prior lymphoid disease and the current HS/DS [40,41,42,43]. One study demonstrated that HS/DS may also possess IGH clonality independently of a pre-existing neoplasm further confounding the understanding of the molecular origins of these diseases [41]. More recently, this has been explained as potential lineage plasticity within the hematopoietic cells [43]. Unfortunately, these studies have thus far failed to further clarify underlying disease mechanisms and tumor origins for HS/DS, necessitating further genomic evaluation.

Several recent studies have evaluated the mutational status of the *BRAF* gene in HS/DS. *BRAF* is a member of the mitogen-activated protein kinase (MAPK) pathway that regulates a series of fundamental mechanisms involved in cellular proliferation, differentiation and apoptosis [44,45,46]. Activating somatic mutations of *BRAF*, most commonly via substitution of valine for glutamic acid at codon 600 (*BRAF* V600E), have been shown to result in constitutive activation of the *MAPK* pathway in several human cancers, including malignant melanomas and thyroid tumors [45]. The presence of the *BRAF* V600E mutation as an initiator of tumorigenesis confers an effective therapeutic target via selective inhibition of the MAPK pathway [44]. Recent studies have shown that ~20% of DS and greater than 50% of HS cases harbor the mutation, providing early evidence that inhibition of the MAPK pathway may offer a promising therapeutic option for these patients [44,45,46]. A mutation orthologous to human *BRAF* V600E was recently described in a series of canine cancers, with the highest frequency in prostatic and urothelial carcinomas (80% and 67% of cases respectively) [47]. Interestingly the mutation was not detected either in canine HM or benign histiocytoma, reducing the likelihood that targeted inhibitors of *BRAF* would be successful in the clinical management of these tumors [47]. Further characterization of *BRAF* sequence and function, and that of its downstream targets, is required to determine whether canine HM and human HS/DS share dysregulation of the MAPK pathway as an underlying mechanism in disease development and progression. Broader mechanistic studies in canine HM may define alternative therapeutic targets for human HS/DS cases, particularly for the 80% of patients with dendritic tumors and 40% with histiocytic tumors that do not harbor the *BRAF V600E* mutation [44,45,46]. 

## 6. Conclusions and Suggestions for Future Directions

Despite several key advances in recent years, canine HM continues to represent a substantial clinical challenge due to the heterogeneity in disease presentation and consequent potential for misclassification, coupled with the absence of broadly effective treatment strategies. Continued efforts are needed to develop more sensitive and specific diagnostic markers that may be applied readily in routine clinical settings. This will require considerable investment in molecular characterization of HM at the genomic, transcriptional and translational level. Since translational dysregulation may offer options for targeted therapy, future studies should include quantitative assessment of members of the pathways highlighted by Boerkamp *et al* [5], particularly those controlling DNA replication and damage repair pathways. These efforts may also reveal the existence of molecular subtypes that explain some of the variation in tumor presentation and clinical behavior, particularly that evident between cases from the two breeds at greatest risk of HM. Molecular profiling will also offer avenues for defining targeted therapies that may offer improved prognoses both for canine and human patients. Treatment resistance and failure are common in canine HM, and the limited number of clinical trials has reduced the ability of the veterinary field to generate a widely accepted standard of care for these patients, despite reports of positive clinical responses in retrospective studies. The rarity of HS/DS in people has similarly stunted progress in the human field. Ultimately maximization of the ability to consider HM in context with its closest counterpart in human patients will be highly dependent on the ability to confirm the cellular origin of the canine disease, and to identify its underlying pathogenic mechanisms. This will determine the degree to which progress in the veterinary field may be translated back to human medicine, as a means to stimulate synergistic advances for these highly understudied diseases.
